# Complete genome sequence of *Cellulomonas flavigena* type strain (134^T^)

**DOI:** 10.4056/sigs.1012662

**Published:** 2010-07-29

**Authors:** Birte Abt, Brian Foster, Alla Lapidus, Alicia Clum, Hui Sun, Rüdiger Pukall, Susan Lucas, Tijana Glavina Del Rio, Matt Nolan, Hope Tice, Jan-Fang Cheng, Sam Pitluck, Konstantinos Liolios, Natalia Ivanova, Konstantinos Mavromatis, Galina Ovchinnikova, Amrita Pati, Lynne Goodwin, Amy Chen, Krishna Palaniappan, Miriam Land, Loren Hauser, Yun-Juan Chang, Cynthia D. Jeffries, Manfred Rohde, Markus Göker, Tanja Woyke, James Bristow, Jonathan A. Eisen, Victor Markowitz, Philip Hugenholtz, Nikos C. Kyrpides, Hans-Peter Klenk

**Affiliations:** 1 DSMZ - German Collection of Microorganisms and Cell Cultures GmbH, Braunschweig, Germany; 2 DOE Joint Genome Institute, Walnut Creek, California, USA; 3 Los Alamos National Laboratory, Bioscience Division, Los Alamos, New Mexico, USA; 4 Biological Data Management and Technology Center, Lawrence Berkeley National Laboratory, Berkeley, California, USA; 5 Lawrence Livermore National Laboratory, Livermore, California, USA; 6 HZI – Helmholtz Centre for Infection Research, Braunschweig, Germany; 7 University of California Davis Genome Center, Davis, California, USA

**Keywords:** non-motile, non-sporulating, aerobic, mesophile, Gram-positive, cellulolytic, xylan degrader, *Cellulomonadaceae*, GEBA

## Abstract

*Cellulomonas flavigena* (Kellerman and McBeth 1912) Bergey *et al.* 1923 is the type species of the genus *Cellulomonas* of the actinobacterial family *Cellulomonadaceae*. Members of the genus *Cellulomonas* are of special interest for their ability to degrade cellulose and hemicellulose, particularly with regard to the use of biomass as an alternative energy source. Here we describe the features of this organism, together with the complete genome sequence, and annotation. This is the first complete genome sequence of a member of the genus *Cellulomonas*, and next to the human pathogen *Tropheryma whipplei* the second complete genome sequence within the actinobacterial family *Cellulomonadaceae*. The 4,123,179 bp long single replicon genome with its 3,735 protein-coding and 53 RNA genes is part of the *** G****enomic* *** E****ncyclopedia of* *** B****acteria and* *** A****rchaea * project.

## Introduction

Strain 134^T^ (DSM 20109 = ATCC 482 = JCM 1489) is the type strain of the species *Cellulomonas flavigena* and was isolated from soil and first described in 1912 by Kellerman and McBeth [1], followed by a description in the first edition of Bergey’s Manual in 1923 [2].

Because of the absence of a definite proof linking the deposited strains to the original description Stackebrandt and Kandler proposed in 1979 *C. flavigena* and six other *Cellulomonas* strains as neotype strains of their respective species [3]. Here *C. flavigena* cells are reported as Gram-positive, non-motile and coryneform with snapping divisions [3].

In addition to the type species *C. flavigena*, the five *Cellulomonas* species, *C. biazotea*, *C. cellasea*, *C. gelida*, *C. fimi* and *C. uda* have been members of the genus since their original description in the first edition of Bergey’s Manual in 1923 [2]. Because of the phenetic resemblance of the different species to each other *C. flavigena* was recognized as the only species in the genus *Cellulomonas* in the eighth edition of Bergey’s Manual. This reduction to a single species was questioned by Braden and Thayer based on serological studies in 1976 [4] and by Stackebrandt and Kandler based on DNA reassociation studies in 1979 [3]. In 1980 the Approved Lists of Bacterial Names already listed six species: *C. flavigena*, *C. biazotea*, *C. gelida*, *C. uda*, *C. fimi* and *C. cellasea* [5]. Currently, 17 species belonging to the genus *Cellulomonas* are noted in the actual version of the List of Procaryotic names with Standing in Nomenclature [6]. Due to the cellulolytic activity of these organisms, their preferred habitats are cellulose enriched environments such as soil, bark, wood, and sugar fields, but they were also successfully isolated from rumen and from activated sludge. Here we present a summary classification and a set of features for *C. flavigena* 134^T^, together with the description of the complete genomic sequencing and annotation.

## Classification and features

The 16S rRNA genes of the 16 other type strains in the genus *Cellulomonas* share between 92.2% (*C. bogoriensis* [7]) and 98.1% (*C. persica* [8]) sequence identity with strain 124^T^, whereas the other type strains from the family *Cellulomonadaceae*, which belong to the genera *Actinotalea, Oerskovia, Paraoerskovia* and *Tropheryma*, share less than 95.6% sequence identity [9]. Cultivated strains with highest sequence similarity include a so far unpublished strain 794 (Y09565) from human clinical specimen (99.7% sequence identity) and Everest-gws-44 (EU584517) from glacial meltwater at 6,350 m height on Mount Everest (98.1% sequence identity). The only reported uncultured clone with high sequence similarity (98.5%) originated from a diet-related composition of the gut microbiota of the earthworm *Lumbricus rubellus* [10]. Metagenomic surveys and environmental samples based on 16S rRNA gene sequences delivered no indication for organisms with sequence similarity values above 93-94% to *C. flavigena*, indicating that members of this species are not abundant in the so far screened habitats. The majority of these 16S rRNA gene sequences with similarity between 88% and 93% originate from marine metagenomes (status June 2010).

**Figure 1:**
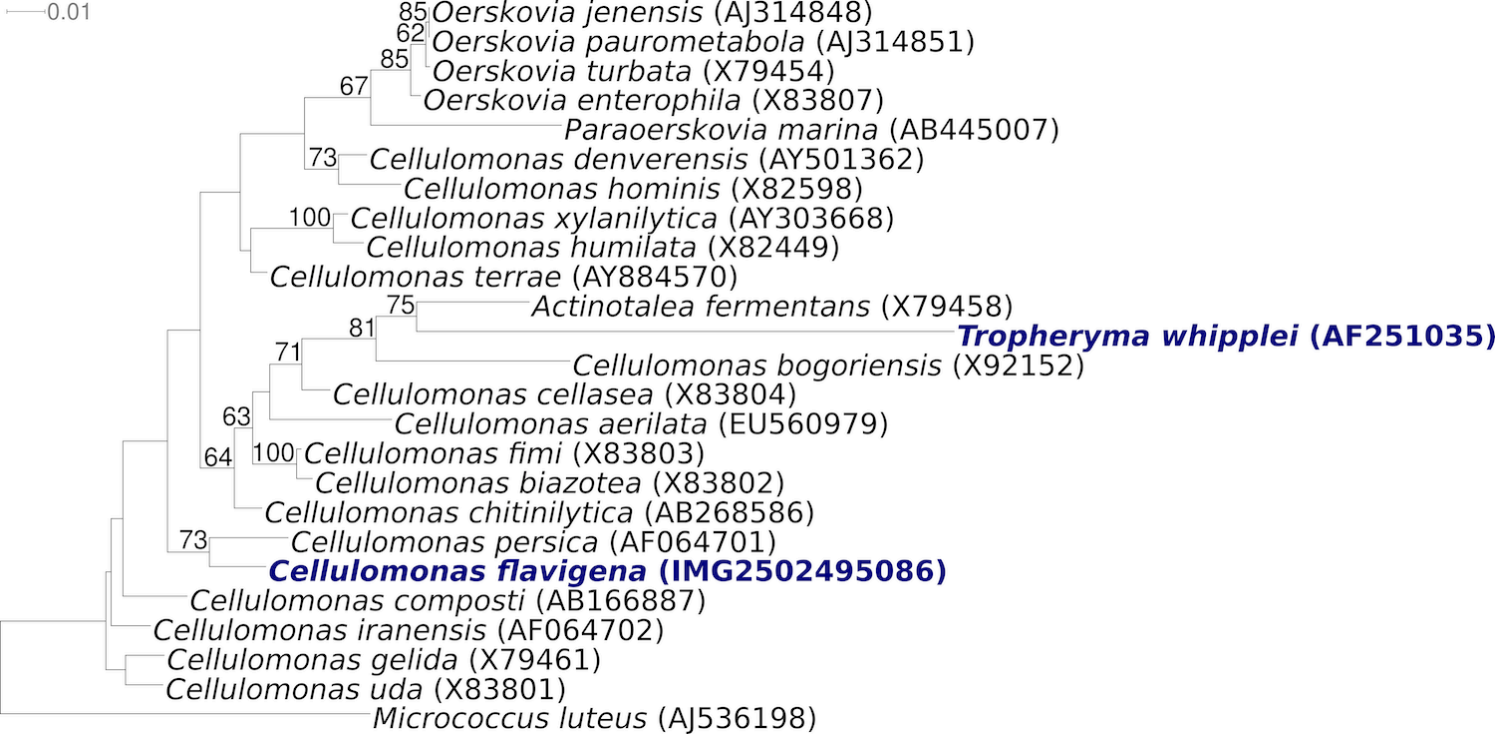
Phylogenetic tree highlighting the position of *C. flavigena* 134^T^ relative to the other type strains within the family *Cellulomonadaceae*. The tree was inferred from 1,393 aligned characters [11,12] of the 16S rRNA gene sequence under the maximum likelihood criterion [13] and rooted with the type strain of the suborder *Micrococcineae*. The branches are scaled in terms of the expected number of substitutions per site. Numbers above branches are support values from 1,000 bootstrap replicates [14] if larger than 60%. Lineages with type strain genome sequencing projects registered in GOLD [15] are shown in blue, published genomes in bold.

Figure 1 shows the phylogenetic neighborhood of *C. flavigena* 134^T^ in a 16S rRNA based tree. The sequences of the two 16S rRNA gene copies in the genome differ by two nucleotides from each other and by up to four nucleotides from the previously published sequence generated from NCIMB 8073 (Z79463).

**Figure 2:**
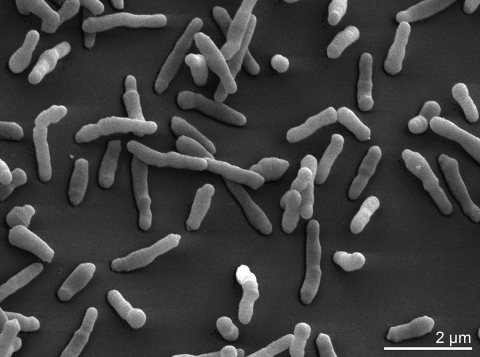
Scanning electron micrograph of *C. flavigena* 134^T^.

Cells of *C. flavigena* stain Gram-positive with a very fast rate of decolorization [3]. Cells in young broth cultures are typically coryneform with a snapping division (Table 1). In week old cultures a transformation to short rods can occur (Figure 2) [3]. On yeast extract-glucose agar *C. flavigena* forms smooth, glistening, yellow colonies about 5 mm in diameter. *C. flavigena* is described as non-motile [3,28], but according to Thayer *et al.* (1984) *C. flavigena* cells possess polar multitrichous flagella [31] (not visible in Figure 2). *C. flavigena* grows under aerobic conditions with an optimal growth temperature of 30°C [2] and an optimal pH of 7 [32].

**Table 1:** Classification and general features of *C. flavigena* 134^T^ according to the MIGS recommendations [16].

**MIGS ID**	**Property**	**Term**	**Evidence code**
	Current classification	Domain *Bacteria*	TAS [17]
		Phylum *Actinobacteria*	TAS [18]
		Class *Actinobacteria*	TAS [19]
		Order *Actinomycetales*	TAS [5,19-21]
		Family *Cellulomonadaceae*	TAS [19,21-25]
		Genus *Cellulomonas*	TAS [5,26,27]
		Species *Cellulomonas flavigena*	TAS [1,5,27]
		Type strain 134	
	Gram stain	positive	TAS [3]
	Cell shape	coryneform with snapping division	TAS [3]
	Motility	non-motile	TAS [3,28]
	Sporulation	non-sporulating	TAS [3]
	Temperature range	mesophile	TAS [2]
	Optimum temperature	30°C	TAS [2]
	Salinity	not reported	
MIGS-22	Oxygen requirement	aerobic	TAS [2]
	Carbon source	fermentation of glucose, maltose, sucrose, xylose and dextrin	TAS [3]
	Energy source	chemoorganotrophic	TAS [3]
MIGS-6	Habitat	soil	TAS [2]
MIGS-15	Biotic relationship	free living	NAS
MIGS-14	Pathogenicity	non pathogenic	NAS
	Biosafety level	1	TAS [29]
	Isolation	from soil	TAS [2]
MIGS-4	Geographic location	not reported	
MIGS-5	Sample collection time	in 1912 or before	NAS
MIGS-4.1 MIGS-4.2	Latitude Longitude	not reported not reported	
MIGS-4.3	Depth	not reported	
MIGS-4.4	Altitude	not reported	

Evidence codes - IDA: Inferred from Direct Assay (first time in publication); TAS: Traceable Author Statement (i.e., a direct report exists in the literature); NAS: Non-traceable Author Statement (i.e., not directly observed for the living, isolated sample, but based on a generally accepted property for the species, or anecdotal evidence). These evidence codes are from of the Gene Ontology project [30]. If the evidence code is IDA, then the property was directly observed by one of the authors or an expert mentioned in the acknowledgements.

Strain 134^T^ is able to ferment glucose, maltose, sucrose, xylose and dextrin, but no fermentation of mannitol was observed [3]. While ribose, acetate and gluconate are utilized, there is no utilization of raffinose and L(+)-lactate [3]. It was shown by Kim *et al.* (1987) that gluconate is catabolized via the Entner-Doudoroff pathway and hexose monophosphate shunt [33]. *C. flavigena* produces catalase but no urease [3]. Esculin and gelatin are hydrolyzed and nitrate is not reduced to nitrite [3].

### Chemotaxonomy

The peptidoglycan of *C. flavigena* contains as the diagnostic amino acid in position 3 of the peptide subunit ornithine with the interpeptide bridge containing D-aspartic acid. The major cell wall sugar is rhamnose, whereas mannose and ribose occur in minor amounts [34]. The major components of the fatty acid profile of *C. flavigena* are 12-methyltetradecanoic (*ai*-C_15:0_) and hexadecanoic (C_16:0_) acids; *i*-C_15:0_, *ai*-C_17:0_, C_14:0_ and C_15:0_ occur in lower amounts [35]. Menaquinone MK-9(H_4_) is the predominant isoprenoid quinone; minor amounts of MK-9(H_2_), MK-8(H_4_) and MK-7(H_4_) were detected [36]. The polar lipids consist of diphosphatidylglycerol, phosphatidylinositol and two so far unidentified phosphoglycolipids [37].

## Genome sequencing and annotation

### Genome project history

This organism was selected for sequencing on the basis of its phylogenetic position [38], and is part of the *** G****enomic* ***E****ncyclopedia of* *** B****acteria and* *** A****rchaea * project [39]. The genome project is deposited in the Genome OnLine Database [15] and the complete genome sequence is deposited in GenBank. Sequencing, finishing and annotation were performed by the DOE Joint Genome Institute (JGI). A summary of the project information is shown in Table 2.

**Table 2:** Genome sequencing project information

**MIGS ID**	**Property**	**Term**
MIGS-31	Finishing quality	Finished
MIGS-28	Libraries used	Two Sanger libraries - 8 kb pMCL200 and fosmids, one 454 pyrosequence standard library and one Solexa library
MIGS-29	Sequencing platforms	ABI3730, 454 Titanium, Illumina GAii
MIGS-31.2	Sequencing coverage	9.1× Sanger; 56.28× pyrosequence
MIGS-30	Assemblers	Newbler version 1.1.02.15, PGA
MIGS-32	Gene calling method	Prodigal 1.4, GenePRIMP
	INSDC ID	CP001964
	Genbank Date of Release	May 13, 2010
	GOLD ID	Gc01326
	NCBI project ID	19707
	Database: IMG-GEBA	2502422318
MIGS-13	Source material identifier	DSM 20109
	Project relevance	Tree of Life, GEBA

### Growth conditions and DNA isolation

*C. flavigena* 134^T^, DSM 20109, was grown in DSMZ medium 92 (Trypticase-Soy-Yeast Extract Medium) [40] at 30°C. DNA was isolated from 0.5-1 g of cell paste using Qiagen Genomic 500 DNA Kit (Qiagen, Hilden, Germany) following the standard protocol as recommended by the manufacturer.

### Genome sequencing and assembly

The genome was sequenced using a combination of Sanger and 454 sequencing platforms. All general aspects of library construction and sequencing can be found at the JGI website (http://www.jgi.doe.gov/). Pyrosequencing reads were assembled using the Newbler assembler version 1.1.02.15 (Roche). Large Newbler contigs were broken into 4,499 overlap ping fragments of 1,000 bp and entered into assembly as pseudo-reads. The sequences were assigned quality scores based on Newbler consensus q-scores with modifications to account for overlap redundancy and adjust inflated q-scores. A hybrid 454/Sanger assembly was made using PGA assembler. Possible mis-assemblies were corrected and gaps between contigs were closed by primer walks off Sanger clones and bridging PCR fragments and by editing in Consed. A total of 704 Sanger finishing reads were produced to close gaps, to resolve repetitive regions, and to raise the quality of the finished sequence. 12,171,379 Illumina reads were used to improve the final consensus quality using an in-house developed tool (the Polisher [41]). The error rate of the completed genome sequence is less than 1 in 100,000. Together, the combination of the Sanger and 454 sequencing platforms provided 65.38× coverage of the genome. The final assembly contains 46,659 Sanger reads and 601,307 pyrosequencing reads.

### Genome annotation

Genes were identified using Prodigal [42] as part of the Oak Ridge National Laboratory genome annotation pipeline, followed by a round of manual curation using the JGI GenePRIMP pipeline [43]. The predicted CDSs were translated and used to search the National Center for Biotechnology Information (NCBI) nonredundant database, UniProt, TIGRFam, Pfam, PRIAM, KEGG, COG, and InterPro databases. Additional gene prediction analysis and functional annotation was performed within the Integrated Microbial Genomes - Expert Review (IMG-ER) platform [44].

## Genome properties

**Figure 3:**
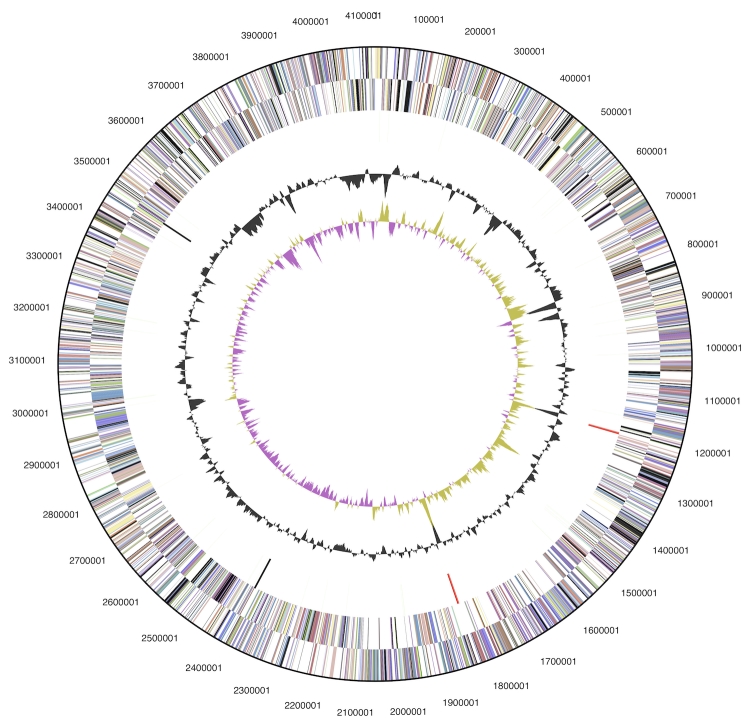
Graphical circular map of the genome. From outside to the center: Genes on forward strand (color by COG categories), Genes on reverse strand (color by COG categories), RNA genes (tRNAs green, rRNAs red, other RNAs black), GC content, GC skew.

The genome is 4,123,179 bp long and comprises one main circular chromosome with a 74.3% G+C content (Table 3 and Figure 3). Of the 3,788 genes predicted, 3,735 were protein-coding genes, and 53 RNAs; 57 pseudogenes were also identified. The majority of the protein-coding genes (71.1%) were assigned a putative function while the remaining ones were annotated as hypothetical proteins. The distribution of genes into COGs functional categories is presented in Table 4.

**Table 3:** Genome Statistics

**Attribute**	**Value**	**% of Total**
Genome size (bp)	4,123,179	100.00%
DNA coding region (bp)	3,725,265	90,35%
DNA G+C content (bp)	3,063,259	74.29%
Number of replicons	1	
Extrachromosomal elements	0	
Total genes	3,788	100.00%
RNA genes	53	1.40%
rRNA operons	6	
Protein-coding genes	3,735	98.60%
Pseudo genes	57	1.50%
Genes with function prediction	2,692	71.07%
Genes in paralog clusters	435	11.48%
Genes assigned to COGs	2,572	67.90%
Genes assigned Pfam domains	2,758	72.81%
Genes with signal peptides	944	24.92%
Genes with transmembrane helices	1,004	26.50%
CRISPR repeats	0	

**Table 4:** Number of genes associated with the general COG functional categories

**Code**	**value**	**%age**	**Description**
J	165	5.8	Translation, ribosomal structure and biogenesis
A	0	0.0	RNA processing and modification
K	270	9.5	Transcription
L	146	5.2	Replication, recombination and repair
B	1	0.0	Chromatin structure and dynamics
D	24	0.9	Cell cycle control, mitosis and meiosis
Y	0	0.0	Nuclear structure
V	64	2.3	Defense mechanisms
T	155	5.5	Signal transduction mechanisms
M	146	5.2	Cell wall/membrane biogenesis
N	8	0.3	Cell motility
Z	0	0.0	Cytoskeleton
W	0	0.0	Extracellular structures
U	38	1.3	Intracellular trafficking and secretion
O	99	3.5	Posttranslational modification, protein turnover, chaperones
C	163	5.8	Energy production and conversion
G	272	9.6	Carbohydrate transport and metabolism
E	209	7.4	Amino acid transport and metabolism
F	85	3.0	Nucleotide transport and metabolism
H	129	4.6	Coenzyme transport and metabolism
I	93	3.3	Lipid transport and metabolism
P	131	4.6	Inorganic ion transport and metabolism
Q	50	1.8	Secondary metabolites biosynthesis, transport and catabolism
R	358	12.7	General function prediction only
S	222	7.9	Function unknown
-	1,216	32.1	Not in COGs

## Insights from genome sequence

A closer look on the genome sequence of *C. flavigena* revealed a set of genes which are probably responsible for the yellowish color of *C. flavigena* cells by encoding enzymes that are involved in the synthesis of carotenoids. Carotenoids are produced by the action of geranylgeranyl pyrophosphate synthase (Cfla_2893), squalene/phytoene synthase (Cfla_2892), phytoene desaturase (Cfla_2891), lycopene cyclase (Cfla_2890, Cfla_2889) and lycopene elongase (Cfla_2888). Cfla_2893 is declared as a pseudo gene, but when ignoring the frame shift the deduced amino acid sequence shows significant similarity to geranylgeranyl pyrophosphate synthases. Geranylgeranyl pyrophosphate synthases start the biosynthesis of carotenoids by combining farnesyl pyrophosphate with C_5_ isoprenoid units to C_20_-molecules, geranylgeranyl pyrophosphate. The phytoene synthase catalyzes the condensation of two geranylgeranyl pyrophosphate molecules followed by the removal of diphosphate and a proton shift leading to the formation of phytoene. Sequential desaturation steps are conducted by the phytoene desaturase followed by cyclisation of the ends of the molecules catalyzed by the lycopene cyclase [45]. It is remarkable that the genes belonging to the putative carotenoid biosynthesis clusters of *Beutenbergia cavernae* (Bcav_3492-Bcav_3488) [46], *Leifsonia xyli* subsp. *xyli* (*crtE*, *crtB*, *crtI*, *crtYe*, *lctB*, *crtEb*) and *Sanguibacter keddieii* (Sked_12750-Sked_12800) [47] have a similar size and show the same organization as in the genome of *C. flavigena*.

In the eighth edition of Bergey’s manual the members of the genus *Cellulomonas* are described as motile by one or a few flagella or non-motile, even within the genus both characteristics occur [32]. Regarding the motility of *C. flavigena* there are different observations described. Thayer *et al.* (1984) report the existence of polar multitrichous flagella [31], whereas Stackebrandt *et al.* (1979) and Schaal (1986) reported *C. flavigena* as non-motile [3,48]. In contrast to Thayer’s observation we found no genes coding proteins belonging to the category ‘flagellum structure and biogenesis’ in the genome sequence. Kenyon *et al.* (2005) report for the genus *Cellulomonas* a coherency between the production of curdlan, a β-1,3-glucan, and non-motility. They observed that the production of curdlan EPS by the non-motile *C. flavigena* leads to a closer adherence to cellulose and hemicellulose. In contrast, cells of the motile *Cellulomonas* strain *C. gelida* produce no curdlan EPS and are not directly attached to the cellulose fibers [28]. The production of curdlan by *C. flavigena* is consistent with the observation of 17 glycosyl transferases (GT) belonging to family 2, as β-1,3-glucan synthases are often found in this GT family.

The characteristic attribute of *C. flavigena* and the other members of the genus *Cellulomonas* is the ability to degrade cellulose, xylan and starch. The most molecular work has been done on cellulase and xylanase genes from *C. fimi*, but also cellulases, xylanases and chitinases of *C. flavigena* were identified and characterized [49-52]. The genome sequence and the subsequent annotation revealed that 9.6% of encoded proteins are classified into the COG category ‘carbohydrate transport and metabolism’. Among them several genes coding for xylan degrading enzymes; 14 genes coding for putative endo-1,4-β-xylanases belonging to glycoside hydrolase family 10 and five genes encoding β-xylosidases. For the hydrolysis of cellulose the concerted action of endo-1,4-β-glucanases, 1,4-β-cellobiohydrolases and β-glucosidases is necessary. Endo-1,4-β-glucanases randomly cleave within the cellulose molecule and increase the number of non-reducing ends which are attacked by 1,4-β-cellobiohydrolases. The released cellobiose is cleaved by β-glucosidases. In the genome of *C. flavigena* two genes coding endo-1,4-β-glucanases (Cfla_0016, Cfla_1897), three genes encoding 1,4-β-cellobiohydrolases (Cfla_1896, Cfla_2912, Cfla_2913) and three genes coding β-glucosidases (Cfla_1129, Cfla_3027, Cfla_2913) were identified.
